# Brain network constraints and recurrent neural networks reproduce unique trajectories and state transitions seen over the span of minutes in resting-state fMRI

**DOI:** 10.1162/netn_a_00129

**Published:** 2020-05-01

**Authors:** Amrit Kashyap, Shella Keilholz

**Affiliations:** Department of Biological Engineering, Georgia Tech and Emory, Atlanta, GA, USA; Department of Biological Engineering, Georgia Tech and Emory, Atlanta, GA, USA

**Keywords:** Networks to dynamics, Brain network models, Neural mass models, Diffusion tensor imaging, Resting-state fMRI, Recurrent neural networks, Generative models

## Abstract

Large-scale patterns of spontaneous whole-brain activity seen in resting-state functional magnetic resonance imaging (rs-fMRI) are in part believed to arise from neural populations interacting through the structural network (Honey, Kötter, Breakspear, & Sporns, 2007). Generative models that simulate this network activity, called brain network models (BNM), are able to reproduce global averaged properties of empirical rs-fMRI activity such as functional connectivity (FC) but perform poorly in reproducing unique trajectories and state transitions that are observed over the span of minutes in whole-brain data (Cabral, Kringelbach, & Deco, 2017; Kashyap & Keilholz, 2019). The manuscript demonstrates that by using recurrent neural networks, it can fit the BNM in a novel way to the rs-fMRI data and predict large amounts of variance between subsequent measures of rs-fMRI data. Simulated data also contain unique repeating trajectories observed in rs-fMRI, called quasiperiodic patterns (QPP), that span 20 s and complex state transitions observed using k-means analysis on windowed FC matrices (Allen et al., 2012; Majeed et al., 2011). Our approach is able to estimate the manifold of rs-fMRI dynamics by training on generating subsequent time points, and it can simulate complex resting-state trajectories better than the traditional generative approaches.

## Author Summary

Brain network models have become a promising theoretical framework for simulating signals that are representative of whole-brain activity such as resting-state fMRI. However, it has been difficult to compare the complex brain activity obtained from simulations with empirical data. Previous studies have used simple metrics to characterize coordination between regions such as functional connectivity. In this manuscript, we extend this work by utilizing modern machine learning techniques to fit the brain network models to observed data and train on the mismatch between the model and observed signal. Our results show that our system training on these new metrics generalizes to a system that is able to reproduce trajectories and complex state transitions seen in rs-fMRI over the span of minutes. Our results will be useful in constraining and developing more realistic simulations of whole-brain activity.

## INTRODUCTION

Over the past decade, our understanding of spontaneous whole-brain activity and coordination between brain regions has largely been obtained through noninvasive resting-state functional magnetic resonance imaging (rs-fMRI) studies (Biswal, Yetkin, Haughton, & Hyde, 1995; Margulies et al., 2016; Smith et al., 2009; Zalesky, Fornito, Cocchi, Gollo, & Breakspear, 2014). Resting state, a state without an explicit task or stimulus, has surprisingly complex whole-brain trajectories that are well structured and highly dependent on the previous brain activity (Allen et al., 2012; Billings et al., 2017; Shakil, Lee, & Keilholz, 2016; Zalesky et al., 2014). Current generative models such as brain network models (BNM) attempt to characterize-whole brain activity as the interaction between a single neural population and the activity of its network neighbors defined by its structural fiber connections as measured through diffusion tensor imaging (Cabral, Hugues, Sporns, & Deco, 2011; Honey et al., 2007). Although there are many variants of the model that use different sets of differential equations to describe the activity at each node, all brain network models heavily rely on the description of the structural network through which they interact (Sanz-Leon, Knock, Spiegler, & Jirsa, 2015).

Long simulations of brain network models, starting from random initial conditions, are able reproduce time-averaged properties of rs-fMRI. These properties such as average functional connectivity (FC) are defined as the correlation between brain regions over long periods of simulated time greater than 10 min (Cabral et al., 2017). The time-averaged properties are thought to be more related to the structural network and are thus able to be reproduced by many different BNMs since they all share the structural network as an input (Cabral et al., 2017; Kashyap & Keilholz, 2019; Skudlarski et al., 2008). However, the BNMs are worse and more variable at reproducing transient dynamic features that occur at shorter timescales, on the order of seconds and minutes, which are much more dependent on the exact description of the differential equations (Cabral et al., 2017; Kashyap & Keilholz, 2019). Since BNMs are not synchronized with actual measurements, there exists a gap in understanding how much these models are able to capture the actual changes to fMRI signal between measurements.

We propose a novel method synchronizing the BNMs to empirical data using recurrent neural networks (RNNs) in order to learn the initial state of the BNM from measured rs-fMRI data. Then using a Euler integration scheme, we can use the differential equations from the BNM to predict the next rs-fMRI data point and then evaluate directly how well it compares against the next measured time point. By applying this technique, brain network autoencoder (BNA), we can quantify how much of the variance of future resting-state activity can be accounted for from previous brain activity using RNNs with BNM constraints as opposed to other sources that influence large brain activity such as external stimuli. This approach of using RNNs with constraints in order to model biological systems has been recently gaining attention as a efficient tool in solving for and modeling unknown systems of differential equations. The approach combines the power of machine learning and allows for the incorporation of known biological variables that allow for interpretation on how the signal evolves (Chen, Rubanova, Bettencourt, & Duvenaud, 2019; Pandarinath et al., 2018). Moreover, the approach has an advantage over traditional methods fitting parameters of the BNM, which simulate over a large parameter space and then use time-averaged measures such as FC for model selection. Rather, the mismatch between the model empirical signal at every time step is fed into the machine learning system to fit model to the data. This approach might also help distinguish between different variants of BNMs as it provides a useful measure in evaluating their performance on short time predictions, as they are all able to produce time-averaged measures as average FC (Cabral et al., 2017).

We evaluate the effectiveness of our model in its accuracy of short-term predictions (< 5 s) that are synchronized to the empirical data and the dynamic properties of the simulated signal over long time intervals (>10 min). In order to train our model, we used fMRI scans from 407 Human Connectome subjects (Van Essen et al., 2013) reduced to 66 regions of interest (ROI) according to the Desikan-Killiany atlas (Desikan et al., 2006). The corresponding structural connectivity to the Desikan-Killiany atlas was estimated using tractrography on five HCP subjects (Kashyap & Keilholz, 2019). After we trained the model, we then evaluate our model on a set of 40 unseen subjects and over 1,000 different initializations to see how well the system generalizes from the training set in order to produce correct predictions on unseen brain activity. We test two variants of this model that have different latent states, the firing rate and the Wilson-Cowan model, in order to see whether this method can distinguish the performance between different variants of brain network models (Cabral, Hugues, Kringelbach, & Deco, 2012a; Deco, Jirsa, McIntosh, Sporns, & Kötter, 2009). We utilize an autoregressive model as a null model to compare our effectiveness on short-term predictions. A similar linear variant (general linear model) is currently being used to distinguish the activity between rest and task blocks and regress out resting-state activity in order to infer task networks (Smith et al., 2009).

We test long periods of simulations of our generative model using dynamic analysis techniques in a similar manner currently used for evaluating traditional BNM (Kashyap & Keilholz, 2019), in order to see whether it can reproduce dynamic properties observed in rs-fMRI that repeat over minutes. We utilize the k-means analysis on short-windowed FCs that looks for structure in the signal in the timescale of around a minute, and a quasiperiodic pattern (QPP) technique that searches for a 20-s repeating pattern. We use a traditional firing rate model as a null model to compare against the long instantiations of BNAs.

The BNA method offers three main strengths in comparison with other methods that are currently used to simulate whole-brain signals:

1.  It solves the problem of comparing simulated and empirical data without using time-averaged metrics such as average FC, by directly using real data to initialize the model and by measuring differences in the predicted transient dynamics on a moment-to-moment basis.
2.  It allows us to use black-box machine learning techniques while simultaneously estimating interpretable latent variables such as firing rate or excitatory and inhibitory currents that can be verified using multimodal recordings.
3.  In long simulations of the BNA, the simulated signal exhibits dynamic properties seen in empirical rs-fMRI that occur over a timescale of minutes, which are not reproducible using traditional BNM techniques.

Therefore, we believe that the brain network autoencoder will be a useful tool to help us understand brain dynamics at the macroscale level.

## RESULTS

### One-Time-Step Prediction

**Figure 1.**
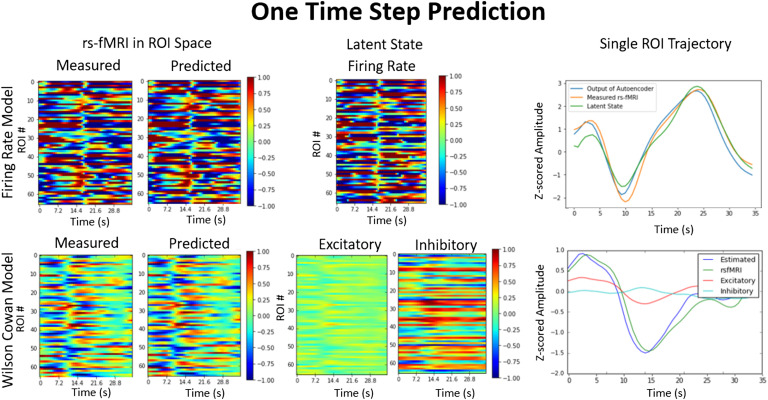
One-step prediction. Two different Brain Network Autoencoders are able to reproduce the next time step from the previous time step. The autoencoder takes as input the measured signal (leftmost) at time step *t* and outputs the predicted (second from left) signal at *t* + 1. The autoencoder projects the input into a space constrained by the brain network model equations (middle panel), which are represented as the state variables in the firing rate model (firing rate) or the Wilson-Cowan model (excitatory and inhibitory currents), and then are integrated to produce the predicted output. The plots represent a 3D contour plot where the x-axis represents time, the y-axis represents the different 66 ROIs, and the color represents the intensity that has been z-scored (see fMRI data subsection in the Methods section). The rightmost panel shows the time series of a single ROI for the input (rs-fMRI), output (estimated), and latent state (firing rate or excitatory/inhibitory currents). At one time step the accuracy in terms of *R*^2^ across all ROIs is on average 0.95.

The sequential autoencoder is trained to predict one time step in advance. In this section we show how the signal is reproduced across 66 regions starting from the input, then projected onto the latent space representing the initial conditions of the BNM, and finally integrated to predict the next time step. In Figure 1, we present the results of predicting the next time step from the previous time step for the two different variants of BNA, the firing rate model and the Wilson-Cowan model. Although both are able to reproduce the spatio temporal signal as shown in Figure 1 (middle top and middle bottom), they differ in the latent or hidden variables used to represent the transitions. For the firing rate BNA, the measured data are projected into a space with firing rate as the hidden variable for each region, as can be seen in Figure 1 (right top). The latent variable time series has a high degree of similarity to the original signal (correlation > 0.9) shown in Figure 1, top right. The latent state is then passed onto the BNM, which integrates it according to the firing rate model to predict the next time-step. The traces of the input, output, and latent state for a single ROI are shown in Figure 1 (left top). For the Wilson-Cowan model, Figure 1 (bottom row), the latent state is represented by two variables, the excitatory and the inhibitory currents, and their interaction through the Wilson-Cowan model produces the next rs-fMRI time step. The excitatory current is positively correlated with the measured signal and the inhibitory current is negatively correlated with the signal, although both to lesser degrees than the firing rate model. The models perform relatively similarly in predicting one time step in front and are able to reproduce the input signal with an *R*^2^ of 0.95 averaged across all areas.

### Multiple-Time-Step Prediction

**Figure 2.**
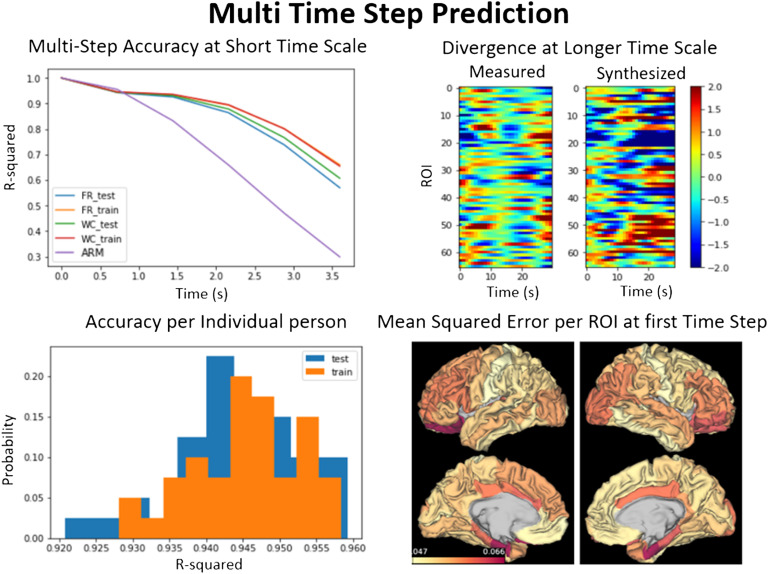
Error across multi-step prediction. Top left: Accuracy of our generative model in synthesizing the first few time points. The accuracy of the firing rate and Wilson-Cowan models are compared on training and test datasets and with the autoregressive model. The error compounds and gradually increases until the model diverges completely from the measured signal around 10 s and continues along its own dynamics (top right). The accuracy over time for the Wilson-Cowan and firing rate training overlap, as both models do about as well as each other on training data. Bottom left: Histogram of *R*^2^ for each individual in test and train datasets shows that it generalizes across individuals. Bottom right: The mean squared error (MSE) for each region of interest (ROI) in predicting the first time step. The MSE is used here to compare differences across ROIs, because it was the error that was used to train the system and is more reproducible across instantiations.

The sequential autoencoder can also predict multiple steps into the future by recursively feeding the predicted output in as the next input. The performance of multi-time-step forecasting is shown in Figure 2 (top left), where the averaged *R*^2^ across a test and a subset of the training data of the same size for both BNA variants are compared with a naive variant of the autoregressive model that assumes the next time point is the previous time point (see Methods). The autoregressive model is similar to the current approach used to differentiate task from rest signals, namely the generalized linear model, which uses the time steps before task activation as a regressor to remove the resting-state activity from task responses (Smith et al., 2009). The generalized linear and the autoregressive model resting state as a constant baseline that does not change over time. Although the autoregressive performs as well as the BNA for the first time point, the BNA is able to reproduce the first three time steps with an *R*^2^ of around 0.9 or higher, as opposed to autoregressive model which is only greater than 0.9 for the first time step. The test and training performance is relatively similar for the autoencoders, only when all the parameters are set correctly and the network is not over- or undertrained (see Methods, Figure 6, for more detail).

Characteristic of autoencoders, the error compounds at every time step, because the previous errors are propagated to the next time step. This causes the model to completely diverge by 10 s from the measured signal, as shown in Figure 2, top right. The bottom left panel in Figure 2 also shows that the BNA generalizes across individuals, as the histogram of the errors is roughly the same for all the individuals in the training or the testing dataset. The two different BNA variants, the firing rate and the Wilson-Cowan, are similar in performance as seen in the top right of Figure 2, with the Wilson-Cowan having on average a higher *R*^2^ on the test dataset. The BNA does not perform equally in predicting each of the ROI time series. It predicts certain regions with a higher accuracy than the others. The mean squared error per each ROI for the first time step is shown in Figure 2, bottom right. The mean squared error was used here instead of *R*^2^, because the network was trained to minimize this gradient during training and most accurately represents the performance on each ROI. The error was largest in the ROIs in the temporal lobe, namely the entorhinal cortex, parahippocampal gyrus, and the temporal pole. These regions are the least connected to the rest of the network and more connected with subcortical regions, which have not been included in the simulations (Cabral et al., 2011).

### Analysis of Long Simulations

**Figure 3.**
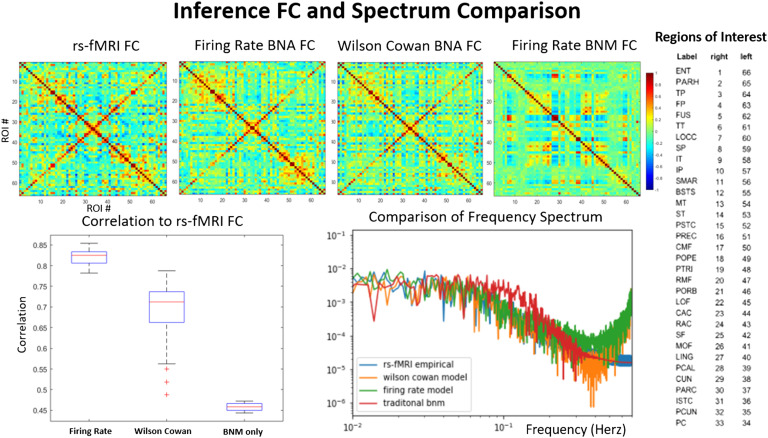
Average functional connectivity and power spectrum. Comparison of average functional connectivity from empirical rs-fMRI (top left), BNA firing rate (top left middle), BNA Wilson-Cowan (top right middle), and a traditional firing rate BNM (top right). The simulated FC matrices have a high degree of correlation (0.7–0.85) with the empirical FC, unlike the traditional BNM that have a correlation of 0.5 (bottom left). Each axis in the FC plots represents the regions in the ROI that are shown on the right. The frequency spectrum (bottom right) of the BNA follows that of the empirical signal exactly except at the higher frequencies (> 0.125 Hz), where the simulated signal has much larger power. The traditional brain network model has less structure in the frequency range (0.01–0.1 Hz) and has equal power in most of the range compared with the rs-fMRI and the BNA models. The traditional BNM and the empirical signal also have been filtered at 0.125 Hz, while the BNA models are not.

In order to assess properties of the simulated signal at longer periods, the BNA was used to generate 1,000 time points or 12 min of data. Properties of longer simulations of BNA were compared with those of the empirical signal. In Figure 3, the average functional connectivity and the power spectrum of the empirical and the BNA as well as a traditional firing rate BNM are compared. The traditional BNM FC has a weak correlation with the empirical FC (0.35) and is in the range of most traditional methods (0.3–0.6; Cabral et al., 2017; Kashyap & Keilholz, 2019; Sanz-Leon et al., 2015; Senden, Reuter, van den Heuvel, Goebel, & Deco, 2017). The BNA performs much better at reproducing the detailed relationship between ROIs seen in FC, compared with the traditional model where groups of ROIs are synchronized over long periods of time, causing blocky patches in the FC when the ROIs are ordered by highly connected subgraphs (Cabral et al., 2011; Kashyap & Keilholz, 2019). The FC of the BNA has a high correlation of 0.83 (firing rate) and 0.7 (Wilson-Cowan) to the actual measured signal. The spectral power of the empirical and the simulated signal is indistinguishable in the range of 0.01 to 0.125 Hz, and has the characteristic 1/f linear slope of around 0.9. The traditional BNM has less temporal structure and is relatively flat over the lower frequency compared with all the other models and the empirical signal (Kashyap & Keilholz, 2019). At higher frequencies the model tends to produce much higher levels of noise than the empirical signal and the traditional BNM, both of which have already been filtered in the preprocessing steps. Before analyzing the simulated signal with dynamical analysis techniques, we therefore filtered it at 0.125 Hz to minimize the high frequency power that would interfere with the dynamic analysis algorithms.

**Figure 4.**
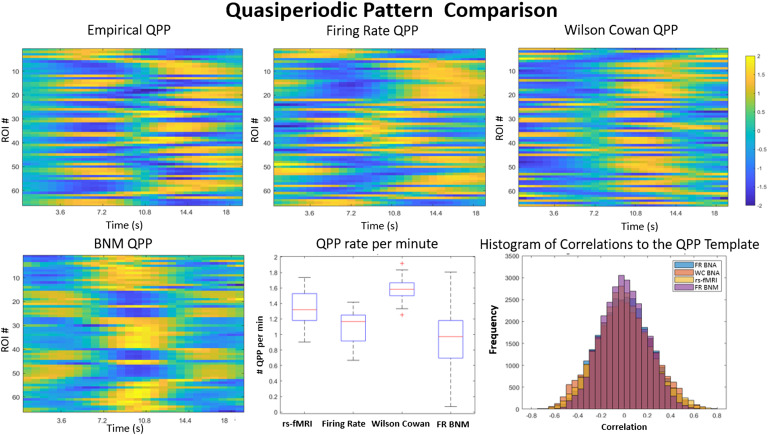
QPP template comparison. Comparison of the different QPP templates is shown in the top row between measured data (top left), the brain network autoencoders (BNA; top middle and right), and the older BNM (bottom left). The QPP templates represent a unique 18-s trajectory of all the ROIs (y-axis) that repeats itself on average 1.3 times per minute (bottom middle). The rs-fMRI signal is highly correlated with the template during specific time points in its trajectory, as seen in the distribution of correlations to the template (bottom right). The Wilson-Cowan and the firing rate BNA have similar distributions, while the BNM template is least correlated with its own data. The firing rate BNA QPP is the closest to the empirical QPP (correlation 0.73) and occurs roughly 1.19 times per minute. The Wilson-Cowan BNA QPP occurs a little faster, around 1.4 times a minute, and has a correlation of 0.43 with the original template. The older BNM QPP is more of an on-off trajectory and does not have the intricate delays and temporal structure as seen in the QPP of the empirical signal or the BNA models.

We also analyzed the simulated signal for unique trajectories known as quasiperiodic patterns (QPP), which could also be considered a limit cycle (Majeed et al., 2011). Limit cycles are a property unique to nonlinear systems, and reproducing such a property would mean that the generative model reproduces some of the dynamics features of rs-fMRI despite its divergence from measured signals. In Figure 4 we have plotted the QPP for the rs-fMRI signal (top left), a traditional generative firing rate BNM model (bottom left), and both of the BNA variants (top middle and right). The empirical QPP involves a 20 s trajectory that switches from task positive networks (first half of the template) to the more internal or default mode networks of the brain (second half of the template; Majeed et al., 2011). After phase adjusting the templates, the maximum correlation of the firing rate BNA QPP was 0.75 and the Wilson-Cowan BNA QPP was 0.43 to the original template. This is very different from the traditional dynamics seen in BNM (bottom left), which produce blocky limit cycles of clusters of nodes that are highly synchronized together and activating together. The BNA produces QPP that are highly structured spatially and temporally. The correlation between the QPP template and the signal is plotted in the bottom middle, where certain time points show a high degree of correlation to the trajectory in the QPP template. Thresholding at 95% significance, the occurrence of these QPP is around 1.3 times a minute in the rs-fMRI data. The BNA models have similar rates, where the firing rate BNA has an occurrence of 1.19 times a minute. The firing rate brain network model shows more variance in the number of QPP cycles per minute (bottom middle, Figure 4).

**Figure 5.**
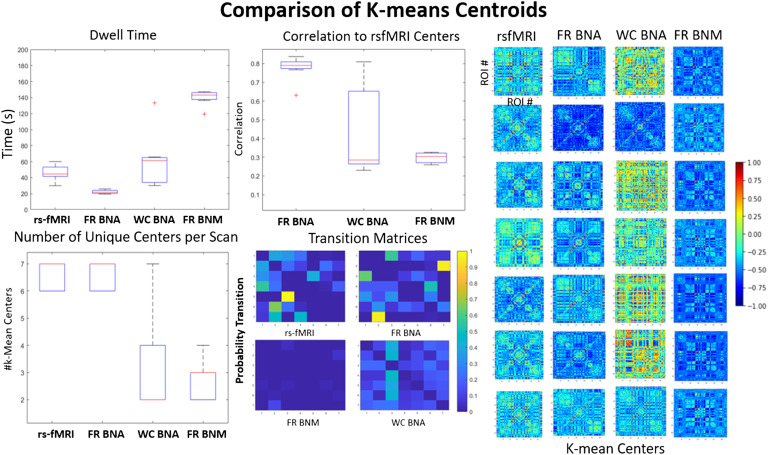
K-means comparison. This figure compares the k-means centers and the transitions for the simulated (BNA and naive BNM) and the empirical signal (30 scans of 15 min). The seven centers are shown in the far right for each category (FR: firing rate, WC: Wilson-Cowan). A boxplot measuring the max correlation is shown (middle top) between each of the simulated centers to the centers from the rs-fMRI data. The dwell time in seconds in each of these centers is shown in the top left. The rest of the transition probabilities (diagonal zeroed out) are shown in the bottom middle. The number of centers in each of the 30 scans is also variable even though they all are defined to have seven clusters across all scans (bottom left).

Another property of rs-fMRI that has been studied is the existence of brain states, which can be described as large scale patterns of functional organization that are stable over the span on the order of around 40 s (Allen et al., 2012; Liu & Duyn, 2013a).The brain transitions through these states over time (Allen et al., 2012). Algorithms such as k-Means have typically identified six or seven states. We applied k-Means clustering on short-windowed functional connectivity matrices (50 s) to find these states in the simulated data (see methods for more detail). In Figure 5, we show the comparison between our BNA models, the firing rate brain network models, and the measured signal for cluster centers as a result of the k-means algorithm. We quantified how close the centers are to each other by taking the maximum correlation of each center to those measured in rs-fMRI. We calculated the length of time in each state (top left), the transition likelihood between states (bottom middle), and how many unique states were observed in a single scan (bottom left). The centers of the BNA models (middle two) compared with the traditional BNM (rightmost) are much more distinct from each other. The Firing Rate BNA model has the highest correlation with the rs-fMRI states (0.8 on average) and a similar number of states seen during a single scan. However, the Firing Rate BNA has a shorter dwell time and seems to move between states faster than observed in the measured signal. The Wilson-Cowan model has more variable and diverse centers and tends to have fewer of them in a single scan, but tends to dwell in them around as long as the measured data. The traditional firing rate brain network model is the least accurate, has few transitions between states, and dwells in a single state for a very long time.

## DISCUSSION

In this manuscript, we adapted the brain network model with the recurrent neural networks in order to make short-time future predictions from observed rs-fMRI. Using this approach, we showed that using the previous measured rs-fMRI data point and an RNN in conjunction with a network-based model, we can predict large amounts of variance in the subsequent time step. We then showed that this system generalizes and can generate trajectories that are similar to resting-state trajectories over larger timescales.

### Predicting Moment-to-Moment Variations

We showed that a network-based model can account for up to 95% of the variance in the fMRI signal between two adjacent time points. This reproduction is not unique, however, and can be estimated using any number of latent variables. Although more complex architectures such as variational autoencoder might be able to successfully predict future rs-fMRI data (Pandarinath et al., 2018), the BNM provides an adequate rough guess of the system dynamics for the autoencoder to converge. This information helps the model to converge during training and make accurate predictions. Moreover, unlike a traditional machine learning approach, this approach yields testable latent variables that can be further evaluated using multimodal datasets, such as magnetoencephalography (MEG) recordings that have been used to generate excitatory and inhibitory currents synchronized with concurrent rs-fMRI recordings (Ritter, Schirner, McIntosh, & Jirsa, 2013).

Fluctuations in spontaneous whole-brain activity have been shown to be nonrandom and highly structured (Zalesky et al., 2014). This suggests that rs-fMRI has both deterministic and stochastic components. The variance explained by the BNA at one time prediction represents a lower bound of the amount of determinism that exists in the signal. It is not surprising that this is the major component of rs-fMRI since the signal has been shown to be highly autocorrelated with itself (Arbabshirani et al., 2014). The simplified first-order autoregressive model, which assumes a steady baseline at the last measured time step, has similar results in performance to the BNA when compared with a single time step and has an *R*^2^ of 0.97. However, for multiple time steps into the future the autoregressive model performs poorly, compared with the BNA models. The two different BNA models perform at short-term scales about as well as each other. This suggests that the trajectory in the short time span is predictable to a certain degree regardless of the approach, but thereafter it starts diverging from the empirical measurements. The divergence from the original trajectory could be due to a number of sources, such as unknown task or stimulus information, noise, not incorporating higher order terms in the BNM, the fallacy of assuming that each ROI behaves in a homogenous fashion, or simply a mismatch between the algorithm and the data that increases over time. Note the BNA itself is not a deterministic system. The latent space variables are modeled as distributions before they are sampled, resulting in a stochastic system.

### Evaluation on Long-Term Dynamics

Although both rs-fMRI and the BNA models are stochastic, long-term simulations of the network-based model are able to reproduce trajectories that are similar to those seen in rs-fMRI. Individual trajectories are varied but they repeat over time, suggesting that rs-fMRI follows a bounded stable manifold that the model is able to estimate. Therefore, random walks across this manifold have shared properties in both the model and the empirical signal. Our results also suggest that most of the resting-state manifold is strongly related to the network-based activity rather than input or random perturbations from noise sources such as higher neural processing.

The strongest metric demonstrating this relationship is average FC, which has a large correlation to the empirical dynamics (0.9 > correlation > 0.8). This is unsurprising since the traditional BNMs do almost as well as the BNAs in this metric, and correlations as high as 0.7 have been reported in the literature (Senden et al., 2017). Average FC seems to be more related to the structural input than the description of the dynamical system (Cabral et al., 2017; Kashyap & Keilholz, 2019). However, the BNA does better than most BNMs in estimating interhemispheric FC correctly, which is usually challenging in network-based models because there are far fewer interhemispheric than intrahemispheric connections detected with diffusion MRI. The power spectrum profile is also mostly reproducible by the model, except in the very high frequency where the model has a lot more power than the empirical signal. This might occur because of the lack of friction in our model, namely that the signals are constantly propagated through feedback loops in the network without loss of energy, unlike the real system. Since most of predictability of the resting state comes from the structured low-frequency activity, we can filter synthesized signal without losing too much information. Other traditional brain network models using the virtual brain have also reported similar performance on power spectrum profiles (Ritter et al., 2013).

Although most traditional BNMs have been able to reproduce to some degree the long-term-averaged properties such as average FC and power spectrum, they have had a harder time in reproducing faster scale dynamics such as reoccurring unique trajectories or the multistate transitions seen in dynamic FC (Cabral et al., 2017; Hansen, Battaglia, Spiegler, Deco, & Jirsa, 2015; Kashyap & Keilholz, 2019). The results from the QPP analysis, which extracts limit cycles, show that the simulated signal has a similar 20-s trajectory and that pattern is repeated over the course of minutes. The results from the k-means analysis on time-varying FC matrices show that the simulated signal has similar state transition in terms of both number and the spatial patterns to those seen in empirical rs-fMRI. This suggests that both of these properties arise naturally in the correct nonlinear network-based representation of rs-fMRI that can be inferred from the data using machine learning techniques. The firing rate BNA seems to fit the data better than the Wilson-Cowan BNA. This might be because the Wilson-Cowan BNA has additional nonlinearities due to the interaction between the excitatory and inhibitory currents.

A direct comparison between our model and other brain network models in the literature on complex dynamical metrics is difficult because most brain network models use their own unique metric to compare against rs-fMRI and there is no established standard. The origin of these complex dynamics has been explained in different theoretical ways. These complex transitions can arise because of the particular nonlinearities of the system (Hansen et al., 2015), which can result in multiple attractors and limit cycles naturally. They can result from parameter changes to the network strength or Hopf bifurcations that cause the system to change its dynamics over time (Deco et al., 2018; Senden et al., 2017). They can also be the result of adding external input and stimuli into the system, causing a change from the zero-input manifold and altering the dynamics (Ashourvan et al., 2019; Deco et al., 2019). These are not mutually exclusive and could induce the changes at once. Our implementation is closest to the first interpretation of rs-fMRI. We explain the observed nonlinear properties of the data purely based on network propagation without the need for external input or a change of a bifurcation variable.

### Errors Across Different ROIs

Using our approach, it is not possible to tease apart the origin of the error that could arise because of a mismatch between the model and the empirical data or because of intrinsic noise. However, looking at the error across regions shows that the error is not evenly distributed across all regions of interest, which can give some clues to where it might arise. The error in reproducing the dynamics at one time step is highest in the nodes of the limbic system (Figure 2, bottom). We believe that our model performs less accurately in this system because they are highly connected to the amygdala and the hippocampus, which are not simulated in the model, and are the least connected nodes to the rest of the network (Cabral et al., 2011). Moreover, tractography has also been known to underestimate the uncinate fasciculus, the major highway between the temporal lobe and the frontal areas, which forms the backbone of the limbic system. The fiber has a very sharp angle that is hard to follow using tractography (Thomas et al., 2014). The echo planar imaging (EPI) sequence used to obtain rs-fMRI data has also known susceptibility issues at interfaces, which would affect the nodes at the proximity such as the frontal pole and the temporal pole, both of which have larger mean squared error compared with the other nodes.

### Comparison With other Machine Learning and Time Forecasting Models

Similar time forecasting has been attempted or is being attempted by several different labs at the time of this manuscript. A variant utilizes a variational autoencoder to find a latent space of brain trajectories that would fit the current data (Brown, Pasquini, Lee, & Seeley, 2019). Another RNN-ICA version uses independento component analysis vectors as the latent space, while another method uses hidden Markov model to model the hidden states (Hjelm et al., 2018; Vidaurre et al., 2018). However, our method is unique in using brain network models as a latent space, whose variables are more interpretable since they represent the state of each neural population’s activity and can be tested using multimodal data. Moreover, none of the other architectures use their model for time series foresting or dynamical analysis, hence their results are not directly comparable to our work, although their methods are similar.

## LIMITATIONS

There are many assumptions that limit the scope of our approach. Machine learning, although good at learning structures in datasets, has a shortcoming of arbitrarily creating a system to fit the data, and every instantiation of the system produces slightly different properties of the simulated system. We tried to address this issue by using various techniques such as using structural constraints, dropout of long short-term memory (LSTM) units, using probability to track the latent variables, and taking the results of multiple runs, in order to make the system more reliable and reproducible. Another limitation of this model is that it needs 50 time points prior to the data point in order to solve for the initial conditions. Shorter time intervals than 50 time points are faster to train, but are less accurate in estimating slow processes. The longer segments required a larger LSTM network and longer training times and were less accurate in our dataset. There are more complex architectures that could solve for the initial conditions faster, such as a forward-backward LSTM architecture (Pandarinath et al., 2018). On the network side, the parcellation scheme reduces the complexity of the signal and discretizes the network. Improvements can be made by allowing for continuous propagation along the cortical sheet, as in the neural field models. Tractography also has its limitations, and better estimates of structural networks should make the model more realistic and improve results especially in regions that are not very strongly connected to the rest of the network. Simulating more of the central nervous system including subcortical regions would also lead to a more biologically plausible model.

## CONCLUSION

We set out to investigate the extent to which network-based theory can explain the moment-to-moment variations seen in rs-fMRI signal’s. Using a novel machine learning approach, we solve for the initial state of traditional network-based models and show that we can account for most of the variation seen in the signal and predict accurately (> 0.6 R^2^) for at least five consecutive time points. Longer instantiations of the system show that our model is able to produce complex trajectories of the nonlinear dynamical system on the order of minutes. We believe that our BNA will be useful when a generative model of rest is needed. Moreover, it can be trained to predict in real time, which allows contrast against dynamics that contain deviations from rest such as in task fMRI studies. In the future, it can also be used to investigate deviations from the manifold such as in task input or due to noisy sources.

## METHODS

### Mathematical Background

**Figure 6.**
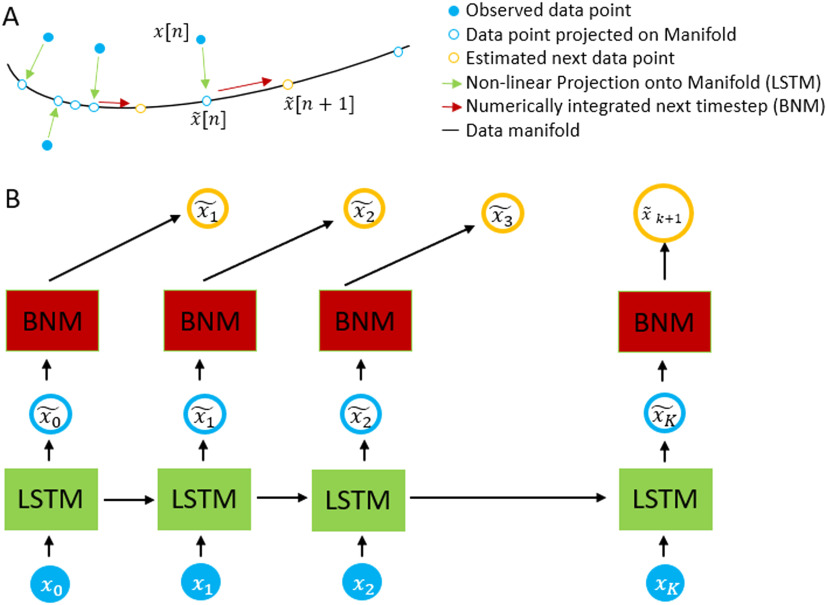
Schematic of the autoencoder. The measurement *x*(*n*) is passed into the LSTM in order to estimate $\tilde{x}$(*n*), which lies in the data manifold. Using the BNM forward equations and $\tilde{x}$(*n*) as our initial conditions, we estimate $\tilde{x}$(*n* + 1). The system is trained by difference in our predicted versus actual measurement at *x*(*n* + 1).

The brain network autoencoder is constructed using the constraints from the brain network model, in conjunction with a recurrent neural network variant known as long short-term memory. The overall design is shown in Figure 6 and implemented using Python TensorFlow. The architecture is a sequential autoencoder, as it is trained with the previous time point to predict the next consecutive time point and uses a latent space where the dynamics are constrained to a smaller space defined by BNM equations to reconstruct the next time point.

Formally, in order to predict the next time point, for each neural measured time point *x*(*n*) we map it to the space *F*(*x*(*n*)). *F* is the transformation performed by the RNN and lives in *R*^*M*×*M*×*T*^, where *M* represents the *M* distinct ROIs being modeled and *T* is the length of previous time points that the LSTM depends on. The next time point is computed as *x*(*n* + 1) = *BNM*(*F*(*x*(*n*))). In essence, the LSTM does a nonlinear coordinate transform of the vector *x*(*n*) into the brain network space where the dynamics are well defined and we can predict the next time point. This process is shown pictorially in Figure 6A, where we show the projection of each data point shown in filled blue circle’s into the manifold represented by the BNM shown in hollow blue circle’s. On the manifold, we can use the BNM equations to update it to the next time step shown in orange. Figure 6B shows the actual architecture used to update the time steps.

For the simplest implementation of BNM, the firing rate model, we can assume the function to be linear with the observation such that *BNM*(*x*) becomes *A* × *x*, where the matrix *A* is the graph Laplacian and *A* = *k* × *SN* − *I*, where *k* < 1 and *SN* is the structural matrix as measured through tractography using diffusion tensor imaging (see Methods section, Experimental Data, Structural network; Hagmann et al., 2008). We use graph Laplacian because it represent’s a well-studied dynamical system known as the consensus equation. On its own, the consensus system does not add in any unstable dynamics because all of its eigenvalues are less than 0, if *k* is set to less than 1 (Mesbahi & Egerstedt, 2010). Therefore the network propagation dies out over subsequent time steps. The eigenvectors of *A* have also shown similarities to rs-fMRI networks (Atasoy, Donnelly, & Pearson, 2016). This algorithm assumes that the Jacobian matrix representing the changes of one brain region with respect to another more or less lies in the direction of the structural fiber network and the nonlinear discrepancies are dealt with by the LSTM (Honey et al., 2007). This can be seen in the Results section (Figure 1), where latent space of the firing rate model is almost identical to the measured data, suggesting that the transformation is near an identity transformation. In a more complex BNM, such as the Wilson-Cowan, the excitatory current is strongly correlated with the signal although less than in the firing rate model, as the model has its own inbuilt nonlinearities and deviates further from the graph Laplacian.

### Implementation

The preprocessed data (see Methods section, Experimental Data, fMRI data) is first cut into contiguous segments of length *k*. This whole segment is then passed into the long short term memory unit as shown in Figure 6B. The units are built using the TensorFlow Python module, specifically the graphics processing unit boosted version to improve speed and performance. The LSTM units take in a series of consecutive time points, and output a sequence of the same length of time points. The LSTM units are a form of recurrent neural networks and have memory of previous time points by using a hidden state vector that it uses as an input to itself for the next consecutive time point. Hence, LSTMs have become popular in the machine learning community because of their success in using this architecture in modeling time series such as speech and natural language processing, in self-driving cars, and even in neural Turing computers thought to emulate biological intelligence (Graves, Mohamed, & Hinton, 2013; Graves & Schmidhuber, 2009; Graves, Wayne, & Danihelka, 2014). Moreover, they solve the problem of learning structure across infinite sequences of consecutive time points by using a forget gate to truncate inputs seen from a long time ago. In practice this means that they need to be trained with a finite sequence length of data.

**Figure 7.**
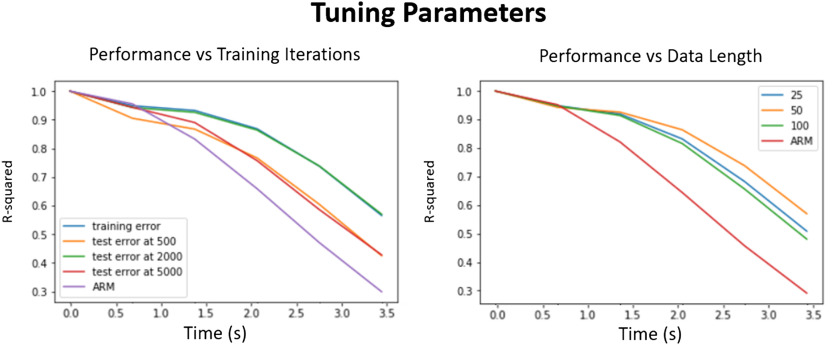
Tuning parameters. Left: The effect of over- and undertraining the network. The performance on the test data compared with the training data at 500 and 5,000 is much worse. For our network size it performed best at around 2,000 iterations. It is compared with the autoregressive model as baseline. Right: The effect of picking different length segments and the performance accuracy. Again the maximum is closer to the middle, which was in our case 50. Too small and too large networks are slightly worse at learning the relationship between past and future rs-fMRI time points.

For our implementation we tested data of length 25, 50, and 100 time points (18, 36, 72 s), as seen in Figure 7, left. The model performed best on 50-length segments, and slightly worse for shorter and longer segments. The LSTM network was also stacked into several layers in a similar manner that convolutional neural networks are stacked together in a series. We used seven identical layers to model the fMRI time series. In general, more layers improve accuracy as long as there is enough data in the training set to scale the size of the network; otherwise, there is a risk on overfitting. Using the inference error as a metric, we also swept the number of training iterations until the performance on unseen cross-validated testing data was about the same as the training data as shown in Figure 7, right. For the cross-validation we split the data of 447 individual scans for 40 test and 407 training samples randomly. At the right amount of training steps, the system does relatively equally in test and training sets. An overtrained or undertrained network, on the other hand, resulted in large differences in test and training, although all three models do equally well on the training dataset. In order to additionally control for overfitting, we also used the inbuilt TensorFlow dropout function that prunes a large number of the weaker weights used in the LSTMs. This has been shown in neural networks to better generalize to unseen test data (Srivastava, Hinton, Krizhevsky, Sutskever, & Salakhutdinov, 2014).

To speed up the training process, we utilized minibatches, where multiple instances of the training data are used simultaneously to train the network (Ioffe & Szegedy, 2015). The number of instances that the network can be trained on simultaneously depends on the size of the training data, and with 400 subjects we used 20 instances to simultaneously train the algorithm. The LSTM network in our model is initialized to a random point, and the first time segment supplies the initial state for the next segment. The performance on the very first block is very poor because of the unknown hidden state and is not included in our evaluation of the algorithm in the Results section. This is a limitation with our implementation, and more complex architectures that solve for the initial state might circumvent this problem.

For our BNM, we choose the firing rate model and the Wilson-Cowan model described in more detail in Box 1. Our choice of brain network models reflects the constraints of this approach. Unlike the traditional brain network model where the simulation time step can be arbitrary, we are constrained by the one-time-step prediction approach of synchronizing with the measured data. Therefore, models that utilize high-frequency oscillators such as the Hopf or the Kuramoto model and are usually simulated at a higher time resolution are more awkward to adapt into the fMRI framework, but would be more useful for training on faster datasets such as MEG or electroencephalogram (EEG). The two different models were also chosen in order to characterize the approach in terms of a simpler linear firing rate model and the more complex multistate nonlinear Wilson-Cowan model. To account for noise in the brain network model, we chose to define the output of the LSTM as a distribution with a mean and standard deviation. We then sample from this distribution in order to generate the initial state. By representing the mapping as a nondeterministic process, the algorithm generalizes to perform better on test datasets and gives more robust results between instantiations.

**Box 1. Brain network models**

**Figure 8.**
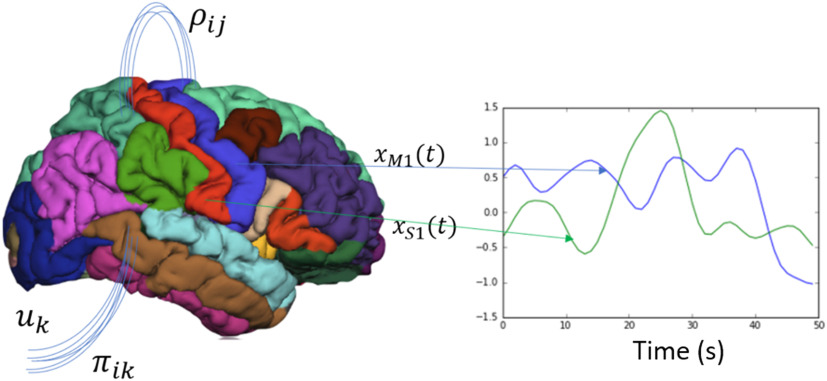
Brain network model. The brain network model state space is constructed by averaging the time courses of each parcellated region. The change of one of those areas *x*_*i*_ is a function of its own activity and its neighbors’ activity that it is connected with *ρ*_*ij*_, and the projection of external cortical input *u*_*k*_ to the brain via *π*_*ik*_.

The brain network model is constructed by specifying a parcellation or atlas, and each region of interest becomes the node and the edges represent the number of fibers between regions and is calculated using tractography. A brain network model in its most general form (see Figure 8) describes the change in neural activity *x* in region of interest *i* as a function of a sum of its neighbors’ *j* activity and its own activity and the physical properties of neural communication between *i* and *j* represented by the vector *ρ* (i.e., the number of fibers between regions, the delay in propagation). The network dynamics are also mediated by a k-dimensional vector *u* representing all subcortical and sensory inputs, and the vector *π* representing again the physical properties that project these inputs into the brain (i.e., thalamic tracts into cortex).

$$\dot{x_{i}}=\underset{j\in Neighbors\;of\;i}{\sum }F(x_{i},x_{j},\rho_{ij})+\underset{k\in Task\;inputs}{\sum }G(u_{k},\pi_{ik})+N(0,\sigma ).$$ (1)

For resting-state activity, the assumption is that *u*_*k*_(*t*) = 0 ∀ *t* and the first term dominates the activity. The function *F* for example, can be as simple as the firing rate model

$$\dot{x_{i}}=-x_{i}+k\underset{j\in Neighbors\;of\;i}{\sum }w_{ij}\times x_{j},$$ (2)

where *w*_*ij*_ represents the number of fibers between *i* and *j*, and *k* represents the global coupling parameter. In a more complex model the state variable *x* can also be represented by multiple variables such as the Wilson-Cowan model shown in the equation below, which uses excitatory and inhibitory currents to describe the change in activity at every region of interest. In the firing rate model the output is taken to be the firing rate, and in the Wilson-Cowan model the fMRI signal is assumed to be just the excitatory signal since it dominates metabolically. These models are thus used to generate whole-brain signals by choosing a random initial point and updating the next step via integration and generating the time series.

$$\dot{E_{i}}=-E_{i}+\alpha \times S(c_{ee}E_{i}-c_{ei}I_{i}+k+\underset{j\in Neighbors\;of\;i}{\sum }w_{ij}\times E_{j}+w_{ij}\times I_{j}).$$ (3)

$$\dot{I_{i}}=-I_{i}+\alpha \times S(c_{ie}E_{i}-c_{ii}I_{i}+k+\underset{j\in Neighbors\;of\;i}{\sum }w_{ij}\times E_{j}+w_{ij}\times I_{j}).$$ (4)

$$S(x)=\frac{1}{1+e^{-x}}$$ (5)

The output of the BNM is taken to be the next fMRI predicted time step. The loss function then is taken as the difference between the predicted and the empirical next time points, and the autoencoder is trained based on this gradient. By forcing the output of the BNM to be the next predicted fMRI signal, the output of the LSTM is forced to become the closest initial time point and the LSTM solves for the nonlinear transformation. We used the TensorFlow Adam Optimizer with a learning rate of 0.0001 to solve for the autoencoder.

### Experimental Data

#### Structural network.

To estimate the structural network we ran tractography on five HCP diffusion-weighted images using the freely available software MRtrix (Kashyap & Keilholz, 2019; Van Essen et al., 2013). From the tractography we estimated the number of fibers that intersected two ROIs in the Desikan-Killiany atlas and normalized the power by dividing by the surface area of the receiving region (Cabral et al., 2011; Desikan et al., 2006). The matrix is finally normalized by dividing by the largest eigenvalue in order for the graph Laplacian (*kSN* − *I*) to have eigenvalues that are all negative (Cabral et al., 2011). This normalizes the dynamics so that the feedback decays over time, and does not exponentially increase the signal over time. The value of *k* is a hyperparameter, but simulations over a few different values around 0.9 showed that it made little difference, because the LSTM would just adjust its output correspondingly. The algorithm is robust as long as it is biased around values that would allow it to converge. For the Wilson-Cowan we set both the *k* values to 0.9 as well, and learned the other parameters. We could also learn the value *k*, but since it is not unique the reproduced latent state ends up further away from the signal. Since the autoencoder will fit the data either way, it is important to determine the constraints from the onset and constrain the latent state to be closer to the measurements.

#### fMRI data.

We trained our algorithm using both resting-state and task data scans. On evaluation, we have only shown our results on testing resting-state data and not task data. This was done because the algorithm was able to able to perform better on our short- and long-term metrics when trained with more varied data such as task. Since the focus of this paper is only on resting state, we have only shown our results on evaluating resting-state metrics on held out resting-state data, but we are planning to address task data in a future publication.

We acquired our data from the 447 minimally processed surface files from the Human Connectome Project (Van Essen et al., 2013). We took the MSMAII scans that were registered to standard space and in CIFTI format and ICA-denoised them utilizing the 300 MELODIC ICA vectors that are provided from HCP. We transform from the surface-voxel time series to the ROI time series by averaging all voxels according to the parcellations established by the Desikan-Killiany atlas. This was done on an individual level since the surface parcellations are provided by HCP and FreeSurfer for each individual subject (aparc and aprac2009 files). The signal is then bandpass filtered from 0.0008 Hz to 0.125 Hz and then global signal regressed using a general linear model with the mean time course of all cortical parcellations. The final signal is subsequently normalized along both axes (Kashyap & Keilholz, 2019). For the task data, each dataset was processed separately (language, working memory, motor, social, emotional, gambling, relational) and then concatenated together. Each task dataset was rounded to the closest multiple of 50 and the autoencoder fed alternating segments of task and rest data. This signal is then fed as both the input and the output to the autoencoder and is the signal that we refer to as the empirical rs-fMRI for the rest of the paper (Kashyap & Keilholz, 2019).

### Dynamical Analysis Techniques

The BNA timeseries were first filtered (0.008–0.125 Hz) before analyzing the properties using dynamical analysis techniques. The dynamical analysis techniques such as QPP and the k-means analysis are described in detail in our previous publication, which outlines metrics in order to compare the simulated whole-brain signal and the rs-fMRI signals (Kashyap & Keilholz, 2019). The QPP algorithm randomly picks a 20-s segment of data and correlates it with the whole signal. At the regions of peak correlation, the algorithm sums up all segments and creates a new template and iteratively converges to a repeating pattern (Majeed et al., 2011). The k-means analysis takes in sliding-windowed (36-s) functional connectivity matrices that are Fisher transformed and clusters them into seven different clusters (Allen et al., 2012). We used an L1 distance to calculate the distance between matrices (Allen et al., 2012). The resulting transitions between clusters was then quantified.

## ACKNOWLEDGMENTS

We would like to thank Dr. Chethan Pandarinath, who provided invaluable help and insight into development and interpretation of our initial brain network autoencoder. We would also like to thank Dr. Christopher Rozell for his insightful discussion on the interpretation of the autoencoder.

## AUTHOR CONTRIBUTIONS

Amrit Kashyap: Conceptualization; Formal analysis; Investigation; Methodology; Resources; Software; Validation; Visualization; Writing - Original Draft; Writing - Review & Editing. Shella Keilholz: Formal analysis; Funding acquisition; Investigation; Methodology; Project administration; Software; Supervision; Writing - Original Draft; Writing - Review & Editing.

## FUNDING INFORMATION

Shella Keilholz, National Institutes of Health (http://dx.doi.org/10.13039/100000002), Award ID: 1R01MH111416. Shella Keilholz, National Institutes of Health (http://dx.doi.org/10.13039/100000002), Award ID: 1R01NS078095. Shella Keilholz, National Science Foundation (http://dx.doi.org/10.13039/100000001), Award ID: 1822606.

## TECHNICAL TERMS

Trajectories: A sequence of observed network activity, where the activity evolves from the initial state to the final state through a well-defined path.
Brain network: A discrete description of the brain consisting of nodes representing neural populations and the connections between them.
Brain network model: A generative model that tries to replicate measured brain activity, by modeling neural populations interacting through the structural brain network.
Structural network: The underlying network consisting of long-distance white matter myelinated cables that connect disparate neural populations.
Functional network: Nodes of the network that act in a coordinated way over a given period of the time.
Recurrent neural networks: A neural network where the last computed output is used as feedback for the next input.
Dynamic analysis techniques: Techniques that characterize transient features of a times series, rather than providing time-averaged measures.
Sequential autoencoders: A deep learning architecture that trains on predicting the next timestep from the last observed timestep.
Manifold: A set of all possible trajectories that originate from the same system and that describe how activity can evolve over time.
Long short-term memory: A specific implementation of a recurrent neural network that is very popular in modeling time series.
